# Continuous-variable Quantum Phase Estimation based on Machine Learning

**DOI:** 10.1038/s41598-019-48551-0

**Published:** 2019-08-27

**Authors:** Tailong Xiao, Jingzheng Huang, Jianping Fan, Guihua Zeng

**Affiliations:** 1 State Key Laboratory of Advanced Optical Communication Systems and Networks, and Center of Quantum Information Sensing and Processing, Shanghai Jiao Tong University, Shanghai, 200240 China; 2 Department of Computer Science, University of North Carolina-Charlotte, Charlotte, North Carolina 28223 USA

**Keywords:** Quantum metrology, Quantum optics

## Abstract

Making use of the general physical model of the Mach-Zehnder interferometer with photon loss which is a fundamental physical issue, we investigate the continuous-variable quantum phase estimation based on machine learning approach, and an efficient recursive Bayesian estimation algorithm for Gaussian states phase estimation has been proposed. With the proposed algorithm, the performance of the phase estimation may be improved distinguishably. For example, the physical limits (i.e., the standard quantum limit and Heisenberg limit) for the phase estimation precision may be reached in more efficient ways especially in the situation of the prior information being employed, the range for the estimated phase parameter can be extended from [0, *π*/2] to [0, 2*π*] compared with the conventional approach, and influences of the photon losses on the output parameter estimation precision may be suppressed dramatically in terms of saturating the lossy bound. In addition, the proposed algorithm can be extended to the time-variable or multi-parameter estimation framework.

## Introduction

Machine learning has become a very promising approach in many fields since it has a strong ability to learn features from large data and subsequently identifies the best strategies with few numbers of trials^1,2^. In particular, integrating machine learning into quantum physics or quantum information has become a very novel approach to exploit the quantum effects or solve the quantum information processing problems^1,3–8^. The quantum phase estimation is an important issue in quantum physics and quantum information^9–12^. It plays significant roles in many fields such as quantum tomography^13–16^, quantum imaging^17^, quantum metrology^18^, quantum communication and computation^19,20^. To this end, many quantum machine learning algorithms for phase estimation have been investigated theoretically and experimentally for the discrete variable quantum state, e.g., the $(|N0\rangle +|0N\rangle )/\sqrt{2}$ (NOON) state^21^, the optimal entangled state^3,5^ and the product states^4^. These works have played an important role in optimizing the phase estimation precision. However, the discrete variable quantum states are highly unstable and not convenient to be prepared and stored especially for the NOON state and the optimal entangle state^22^. The measurement scheme of these proposals relies on the high efficient single photon detector. Moreover, the state-of-art machine learning algorithms for quantum phase estimation such as Particle Swarm Optimzation-based algorithm^5^ and Differential Evolution-based algorithm^3^ present a high complexity and the availability of the algorithms is limited by the average photon numbers. These current difficulties hinder the further precision improvement of discrete variable quantum phase estimation and the wide application of it.

One class of quantum states of particular interest is the Gaussian states^23^, which is a kind of continuous-variable (CV) quantum states since they are easy to produce and comparatively robust against losses. For the CV quantum phase estimation in a conventional way, numerous works have been presented. For example, a Bayesian inference-based quantum phase estimation scheme is proposed to achieve the Heisenberg limit (HL) in ref.^24^. In ref.^25^, a theoretical HL scaling scheme with two-mode squeezed vacuum state (TMSV) and parity measurement are put forward. In ref.^26^, an adaptive feedback scheme based on parity measurement and TMSV is studied, which can achieve the HL scaling in phase interval of [0, *π*]. In addition, with prior knowledge^27,28^ of the parameter, ref.^29^ analyzes the Van Trees Bound (VTB) based on Van Trees inequality, which is proven to have higher precision compared with Carmér-Rao Bound (CRB)^30^.

In this paper, we attempt to obtain a general physical model of quantum phase estimation. We take advantages of the practical benefits of CVs by proposing a machine learning algorithm in infinite-dimensional systems. Thus, a novel CV quantum phase estimation based on machine learning has been presented. Based on the proposed algorithm, we perform the Monte Carlo simulations and some interesting performances have been obtained. Some physical problems such as the influence of the photon loss, the range limitation of the phase parameter to be estimated can be solved well with the proposed algorithm.

The remainder of this paper is organized as follows. In Sec. 2, we present a continuous-variable quantum machine learning algorithm to phase estimation based on the Mach-Zehnder interferometer and the recursive Bayesian estimation theory. In Sec. 3, we present a performance analysis based on the proposed algorithm for various input Gaussian states, and the effects of the prior information and the photon loss are investigated. The results are summarized in Sec. 4.

## Machine Learning for Quantum Phase Estimation

### Physical model

**Figure 1:**
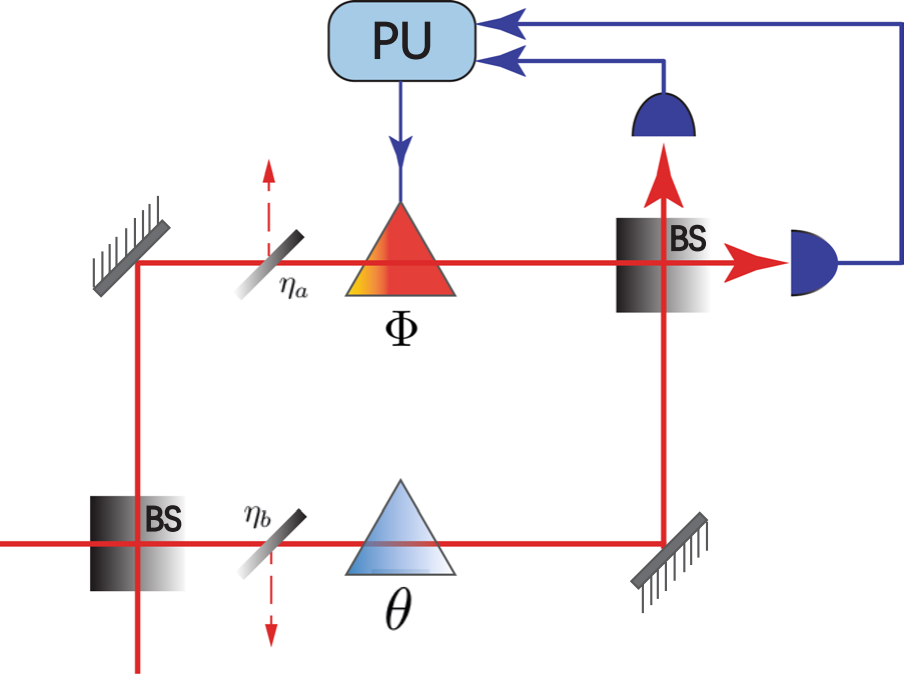
Mach-Zehnder interferometer schematic with an unknown phase difference *θ* between the two arms and an additional controllable phase shifter Φ. The measurement apparatus is chosen to be balanced homodyne detection. The 50:50 beam-splitter is fictitious with transmissivity *η*_*a*_ in upper arm and *η*_*b*_ in lower arm.

Interferometry is an important tool for estimating phase shifts^31^. An interferometer is a physical apparatus that encodes the value of a parameter into a probe state, also called input state. In optical interferometers, phase shifts are generally induced by a lapse in the relative time taken by the light to travel down two distinct paths (the interferometer arms)^25^. Among the common interferometers such as Michelson-Morley interferometer, Sagna interferometer and Mach-Zehnder interferometer (MZI), they are the effective and equivalent physical models to study the phase estimation problem^32–34^. However, MZI is a relatively representative one and generally investigated for its mathematical simplicity in describing the the physical process^32^. Therefore, without loss of generality, we employ the MZI to study the parameter model as Fig. 1 shows. In our physical model, MZI consists of two beam splitters (BS), which are assumed to be 50:50, with two arms from the first BS to the second where the difference in the optical lengths is denoted by the phase shift *θ*. At the output of the second BS are placed by the balanced homodyne detectors (HD) and the single HD will be used to measure the output state. The detection outcomes are transmitted to the processing unit (PU) to obtain the feedback phase Φ by executing machine learning algorithm. All the linear losses are equivalent to the photon loss in two arms, denoted by the loss rate of ${\eta }_{a},{\eta }_{b}$.

We regard Gaussian states as input, and their Wigner characteristic function is given by,

1 $$\chi (\xi )={\rm{Tr}}[\hat{\rho }\,\exp \,(i{\xi }^{T}\hat{R})]=\exp (-\frac{1}{2}{\xi }^{T}{\rm{\Sigma }}\xi +i{d}^{T}\xi ),$$

where $\xi \in {{\mathbb{R}}}^{4}$, $\hat{R}={[{\hat{x}}_{a},{\hat{p}}_{a},{\hat{x}}_{b},{\hat{p}}_{b}]}^{T}$ is the quadrature operator, $\hat{\rho }$ is the density matrix of CV quantum state, ∑ is the second order moment of quantum states $\hat{\rho }$, and *d* is the first-order moment. The employed $\hat{x}$ and $\hat{p}$ denote the position and momentum operators, respectively. We note here, ${\hat{x}}_{\ell }=(\hat{\ell }+{\hat{\ell }}^{\dagger })/\sqrt{2},{\hat{p}}_{\ell }=(\hat{\ell }-{\hat{\ell }}^{\dagger })/\sqrt{2}i,$ where $\ell =a,b$ denotes two modes in MZI and the $\hat{a}(\hat{b}),{\hat{a}}^{\dagger }({\hat{b}}^{\dagger })$ are the creation and annihilation operators with respect to two modes. While the (*i*, *j*)th entry of the ∑ is given by,

2 $${{\rm{\Sigma }}}_{ij}=\frac{1}{2}\langle {\hat{R}}_{i},{\hat{R}}_{j}\rangle -\langle {\hat{R}}_{i}\rangle \langle {\hat{R}}_{j}\rangle ,$$

where 〈·〉 is the expectation of an operator. The *i*th term of *d* is given by,

3 $${d}_{i}={\rm{Tr}}(\hat{\rho }{\hat{R}}_{i}).$$

The corresponding Wigner function *W*(*X*) of pure Gaussian state is given by^35^

4 $$W(X)=\frac{\exp [-\frac{1}{2}{(X-d)}^{T}{{\rm{\Sigma }}}^{-1}(X-d)]}{{(2\pi )}^{2}\sqrt{{\rm{\det }}({\rm{\Sigma }})}},$$

where each of component in $X={[{x}_{a},{p}_{a},{x}_{b},{p}_{b}]}^{T}$ corresponds to the eigenvalue of the operator $\hat{R}$. The Wigner function of the input state is denoted by *W*(*X*_in_). After the input state propagate through MZI, the output state can also be denoted with Wigner function $W({X}_{{\rm{out}}}|\theta ;{\rm{\Phi }})$ with phase shift by Eq. (4), where *X*_out_ is the eigenvalue vector corresponding to the quadrature operator of the output state. Detailed evolution of first-order and the second order moment of Wigner function corresponding to the input and output state are presented in Appendix (see supplementary material). After measuring the optimal quadrature in one output, the corresponding outcome *x* has a probability distribution ${\mathscr{P}}(x|\theta ;{\rm{\Phi }})$ which is given by the marginal integral of the Wigner function over the conjugate quadrature^36^ for one output port

5 $${\mathscr{P}}(x|\theta ;{\rm{\Phi }})=\mathop{\int }\limits_{{\mathbb{R}}}W({X}_{{\rm{out}}}|\theta ;{\rm{\Phi }}){\rm{d}}p.$$

This can be generalized to the situation of partially homodyning a multimode bosonic system.

### Recursive Bayesian inference for phase estimation

The input of algorithm consists of the CV quantum probe states |CV〉, the number of HDs *M*, the prior distribution ${\mathscr{P}}(\theta )$, the transmissivity of BS ${\eta }_{a},{\eta }_{b}$, and the feedback learning rate *λ*. If no prior information is obtained, ${\mathscr{P}}(\theta )$ is set to be a flat distribution. The algorithm is flexible because it adapts to different phase estimation situations by setting the different composition of input parameters as Algorithm 1 presented.

Given an output data set ${\mathscr{X}}=\{{x}_{1},{x}_{2},\cdots ,{x}_{k}\}$, the posterior probability for phase shift *θ* obeys the Bayes formula for which ${\mathscr{P}}(\theta |{\mathscr{X}})={{\mathscr{N}}}^{-1}{\mathscr{P}}({\mathscr{X}}|\theta )\pi (\theta ),$ where ${\mathscr{N}}=\int {\mathscr{P}}({\mathscr{X}}|\theta )\pi (\theta ){\rm{d}}\theta$ is the normalized quantity to legalize the posterior probability distribution, *π*(*θ*) denotes the prior probability. Compared with maximum likelihood estimation (MLE), using the Bayesian method mainly has two merits^2^: (1) Prior information assists one ignore the impossible values the parameter would take. A long tail prior is also beneficial for us to estimate the unknown true phase shift; (2) Bayesian approach uses a set of possible *θ* generated by posterior and adopts the likelihood as a weight to estimate the parameter rather than using single estimation value of *θ*. Hence, Bayesian estimation fully utilizes the prior information to estimate the parameter faster. In particular, recursive estimation is applied in adaptive control, where the parameters of a dynamical system working in a closed loop are tracked using measurements of system input and output. We present a recursive estimation policy, where the transition kernel is an identity function since we assume the phase shift is invariant with time. In fact, recursive Bayesian estimation (RBE) is developed by applying the Bayesian view of statistical inference in general to the recursive estimation problem in particular. Moreover, RBE can utilize the outside feedback to automatically adjust the parameter in order to make the optimal policy. By recursive or reinforcement learning from the outside feedback phase, the optimal parameter estimation will finally be learned. However, the standard Bayesian inference cannot make full use of the feedback phase recursively to estimate the parameter.

The likelihood for *k*th HD is ${\mathscr{P}}({x}_{k}|\theta ;{{\rm{\Phi }}}^{(k)})$, where *x*_*k*_ is the *k*th measurement outcome, and Φ^(*k*)^ is the *k*th feedback phase. The conceptual solution of this RBE for *k* number of measurements is given by,

6 $${\mathscr{P}}(\theta |{{\mathscr{X}}}_{k};{{\rm{\Xi }}}_{k})=\frac{{\mathscr{P}}({x}_{k}|\theta ;{{\rm{\Phi }}}^{(k)}){\mathscr{P}}(\theta |{{\mathscr{X}}}_{k-1};{{\rm{\Xi }}}_{k-1})}{{\mathscr{P}}({x}_{k}|{{\mathscr{X}}}_{k-1};{{\rm{\Xi }}}_{k-1})},$$

where

7 $${\mathscr{P}}({x}_{k}|{{\mathscr{X}}}_{k-1};{{\rm{\Xi }}}_{k-1})=\mathop{\int }\limits_{{\mathbb{R}}}{\mathscr{P}}({x}_{k}|\theta ;{{\rm{\Phi }}}^{(k)}){\mathscr{P}}(\theta |{{\mathscr{X}}}_{k-1};{{\rm{\Xi }}}_{k-1}){\rm{d}}\theta ,$$

is the overall probability used to normalize the distribution. The recursion is initialized with ${\mathscr{P}}(\theta |{{\mathscr{X}}}_{-1})=\pi (\theta )$. Note that ${{\rm{\Xi }}}_{k}={[{{\rm{\Phi }}}^{(1)},{{\rm{\Phi }}}^{(2)},\cdots ,{{\rm{\Phi }}}^{(k)}]}^{T}$ and ${{\mathscr{X}}}_{k}={[{x}_{1},{x}_{2},\cdots ,{x}_{k}]}^{T}$ are the columns of historical feedback phase and measurement outcomes for *k* number of measurements. The recursive process is demonstrated by line 3 to 14 in Algorithm 1, where ${d}_{\eta }^{{\rm{o}}(k)},{{\rm{\Sigma }}}_{\eta }^{{\rm{o}}(k)}$ denotes the *k*th first and second order moment of the quantum state before (*k* + 1)th measurement, respectively. In particular, line 6∼8 implies a quantum physical interaction process, and costs constant number of operations *O*(*K*) in one iteration, where *K* is a constant value. The complexity for evaluation of the posterior distribution is a constant quantity denoted by *O*(*K*) when given the likelihood function and prior distribution. However, the exact normalization requires dividing the phase range into the finer grid which needs at least reaching the ideal precision level of VTB, otherwise the ideal precision can never be obtained under a rough grid (i.e., resolution is less than the ideal precision). Then the normalization process needs to sum over all possible phases within the grid to calculate the normalization constant ${\mathscr{N}}$. Hence, the complexity of exact normalization is inversely proportional to the ideal phase precision *δ*, namely $O({\delta }^{-1})$ for each iteration.

We further design a strategy ${\rm{\Gamma }}({\mathscr{X}})$ to extract the information about *θ* from ${\mathscr{X}}$. ${\theta }_{\ast }^{(k)}={\rm{\Gamma }}({{\mathscr{X}}}_{k})$ is denoted as the *k*th extracted phase information from *k* number of measurements. Then, we adopt an updating approach for which ${{\rm{\Phi }}}^{(k)}=(1-\lambda ){{\rm{\Phi }}}^{(k-1)}+\lambda {\theta }_{\ast }^{(k)},$ where $\lambda \in [0,1]$, Φ^(*k*)^ is the feedback phase for *k*th iteration. This is a common learning (updating) strategy in machine learning algorithms. If *λ* = 0, it is equivalent to the case of no feedback control. The feedback process is demonstrated by line 12 and 13 of Algorithm 1. Intuitively, the feedback phase Φ will approach true phase shift *θ* by our strategy. Note that if the measurement samples are small and Φ cannot be updated adequately towards to *θ*, which would cause a large oscillation at the final updating stage. Thus, as a thumb of rule, the chosen magnitude of *λ* is fairly matched with the measurements trials *M* to render the posterior distribution converge to a correct stationary distribution. The feedback process only needs to calculate the explicit function such that costs *O*(*K*) in one iteration. Thus, the computation complexity of our algorithm is *O*(*Mδ*^−1^). Thus, if we input the coherent states, the ideal precision is the standard quantum limit (SQL) scaled by $\delta ={(MN)}^{-1/2}$ where *N* is the average photon number. If we input the quantum state, the ideal precision is HL scaled by $\delta ={M}^{-1/2}{N}^{-1}$. Therefore, the computation complexity of our algorithm is *O*(*Mδ*^−1^). More specifically, the cost for SQL and HL is $O(\sqrt{{M}^{3}N})$ and $O(\sqrt{{M}^{3}}N)$, respectively. Note that the normalization overhead of Algorithm 1 can be further reduced by using the technique of sampling methods^37,38^.

**Algorithm 1:**
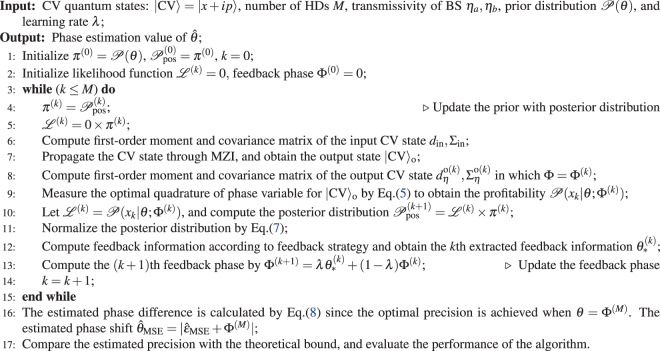
RBE for CV quantum phase estimation.

We adopt the minimum mean-square error (MSE) as the benchmark to evaluate the precision performance. The output is the phase estimation value ${\hat{\theta }}_{{\rm{MSE}}}$ which can be calculated by ${\hat{\theta }}_{{\rm{MSE}}}=|{\hat{\varepsilon }}_{{\rm{MSE}}}+{{\rm{\Phi }}}^{(M)}|,$ where Φ^(*M*)^ is the *M*th (ultimate) feedback phase and ${\hat{\varepsilon }}_{{\rm{MSE}}}$ is the ultimate phase difference (i.e., $\varepsilon =\theta -{\rm{\Phi }}$) between two arms of MZI. Ideally, if ${\hat{\varepsilon }}_{{\rm{MSE}}}=0$, the visibility of MZI will be the highest, and then we capture the most accurate phase measurement. The phase difference ${\hat{\varepsilon }}_{{\rm{MSE}}}$ is calculated by,

8 $${\hat{\varepsilon }}_{{\rm{MSE}}}=\int \hat{\varepsilon }{\mathscr{P}}(\varepsilon |{{\mathscr{X}}}_{M};{{\rm{\Xi }}}_{M}){\rm{d}}\hat{\varepsilon }.$$

To analyze the optimal performance of MSE benchmark, we assume a general estimator ${\rm{\Theta }}({\mathscr{X}})$, and it offers a necessarily imperfect estimation of the phase difference *ε*. Here, we present a Bayesian CRB suitable for finite samples with Van Trees inequality. The VTB in our physical model is given by,

9 $$\begin{array}{ll}{{\mathscr{V}}}^{2}[{\rm{\Theta }}({\mathscr{X}});\varepsilon ;\pi ;{\rm{\Xi }}] & =\iint {\rm{d}}{\mathscr{X}}{\rm{d}}\varepsilon \pi {\mathscr{P}}({\mathscr{X}}|\varepsilon ;{\rm{\Xi }}){({\rm{\Theta }}({\mathscr{X}})-\varepsilon )}^{2},\\  & \ge {{\mathscr{K}}}^{-1}(\pi ;{\rm{\Xi }}),\end{array}$$

with

10 $$\begin{array}{ll}{\mathscr{K}}(\pi ;{\rm{\Xi }}) & =\iint {\rm{d}}{\mathscr{X}}{\rm{d}}\varepsilon \{{\mathscr{G}}[{\mathscr{P}}({\mathscr{X}}|\varepsilon ;{\rm{\Xi }})]+{\mathscr{G}}[\pi ]\}{\mathscr{P}}({\mathscr{X}}|\varepsilon ;{\rm{\Xi }})\pi \\  & =\,{\mathbb{E}}[ {\mathcal I} (\varepsilon ;{\rm{\Xi }})]+ {\mathcal I} (\pi ),\end{array}$$

where ${\mathscr{G}}[\alpha (\theta )]={({\partial }_{\theta }\mathrm{log}\alpha )}^{2}\alpha$, ${\mathcal I} (\,\cdot \,)$ is the Fisher information^39^, ${\rm{\Xi }}$ and ${\mathscr{X}}$ denote the set of feedback phase and measurement outcomes. Note that if the prior distribution is flat, the VTB equals to CRB. In general, we assume an optimal Gaussian prior of width *σ* > 0 centered at $\bar{\theta }={\theta }_{p}$. The width of Gaussian prior *σ* needs to be restricted within an interval since a large width will cause the MSE rule not working well for phase estimation. More specifically, the Gaussian prior width needs at least half of the interval of *θ* to guarantee the efficiency^40^. For a quantum phase estimation system, the quantum CRB (QCRB) is given by^41,42^,

11 $${{\mathscr{V}}}^{2}[{\rm{\Theta }}({\mathscr{X}});\theta ]\ge \frac{1}{M {\mathcal F} (\theta )}.$$

QCRB establishes that apart from the statistical scaling (*M* is the number of measurements), the variance of any unbiased estimator of the phase shift is bounded by the inverse of the quantum Fisher information ${\mathcal F} (\theta )$^30^. In our physical model, all states and operations are Gaussian, thus ${\mathcal F} (\theta )$ can be calculated by^43^,

12 $${\mathscr{F}}(\theta )={(\frac{{\rm{\partial }}d}{{\rm{\partial }}\theta })}^{T}{{\rm{\Sigma }}}^{-1}\frac{{\rm{\partial }}d}{{\rm{\partial }}\theta }+{\rm{T}}{\rm{r}}[{(\frac{{\rm{\partial }}{\rm{\Sigma }}}{{\rm{\partial }}\theta }{{\rm{\Sigma }}}^{-1})}^{2}].$$

The QCRB is a useful tool to qualify the performance of an interferometer since it applies to all possible quantum measurements on the output modes. Our RBE-based machine learning algorithm develops an integral framework for quantum phase estimation to fast saturate theoretical precision using limited number of measurements for CV quantum phase estimation.

### Performance analysis

#### Coherent state without feedback

According to the proposed algorithm, we investigate the phase estimation problem via Monte Carlo simulation with different typical Gaussian states. We consider a classical scenario where the coherent state $|\alpha \rangle \otimes |0\rangle =\hat{D}(\alpha ,{\theta }_{0})|0\rangle \otimes |0\rangle$ is regarded as input state. $\hat{D}(\alpha ,{\theta }_{0})$ is the displacement operator, and its matrix form in phase space is given by $D(\alpha ,{\theta }_{0})={\rm{diag}}\{|\alpha |\,\cos \,{\theta }_{0},|\alpha |\,\sin \,{\theta }_{0}\}$, where *θ*_0_ is the displacement angle. Let *θ*_0_ = 0 implying that displace the vacuum state right along the *x*-axis in phase space. As for the coherent state, the input Wigner function is given by,

13 $$W({X}_{{\rm{in}}})=\frac{\exp [-{(X-{d}_{c})}^{T}{{\rm{\Sigma }}}_{c}^{-1}(X-{d}_{c})]}{{(2\pi )}^{2}\sqrt{{\rm{\det }}({{\rm{\Sigma }}}_{c})}},$$

where ${{\rm{\Sigma }}}_{c}=\frac{1}{4}{{\mathbb{I}}}_{4}$ is the covariance matrix and ${d}_{0}={\{|\alpha |,0,0,0\}}^{T}$ is the mean vector. When the coherent state passes through the lossless MZI (${\eta }_{a}={\eta }_{b}=1$), the first-order moment and covariance matrix of the output state are calculated, which is given by ${d}_{{\rm{opc}}}=\frac{1}{2}|\alpha |{\{\cos \varepsilon ,-\sin \varepsilon ,\sin \varepsilon ,\cos \varepsilon \}}^{T}$ and ${{\rm{\Sigma }}}_{{\rm{opc}}}=\frac{1}{4}{{\mathbb{I}}}_{4}$. *ε* = *θ* − Φ is the phase difference between two arms. Here, we have ignored the global phase difference since it does not impact our results of phase estimation. In this part, let Φ = 0, *σ* = *∞* because we do not consider the feedback control and prior information in our algorithm.

The quadrature ${\hat{x}}_{\psi }=\frac{1}{2}({e}^{-i\psi }\hat{a}+{e}^{i\psi }{\hat{a}}^{\dagger })$ is measured by means of HD on repeated input state. Here, we find the quadrature $\hat{x}$ measurement for second output port is optimal, thus let *ψ* = 0 and the probability of obtaining *x* for single measurement given by,

14 $${{\mathscr{P}}}_{c}(x|\theta )=\sqrt{\frac{\pi }{2}}\exp \{-2{(x-\frac{|\alpha |}{2}\sin \theta )}^{2}\},$$

where $|\alpha {|}^{2}$ denotes the average photon number. The Fisher information of Eq. (14) ${ {\mathcal I} }_{c}(\theta )=|\alpha {|}^{2}{\cos }^{2}\theta$. Thus, the VTB of using HD for *M* independent identical measurements is ${(\delta \theta )}_{H}^{2}=1/(M|\alpha {|}^{2}{\cos }^{2}\theta )$. Obviously, when ${\cos }^{2}\theta =1$, i.e., ${\theta }_{{\rm{opt}}}=k\pi ,k\in {\mathbb{Z}}$, the precision ${(\delta \theta )}_{{\rm{opt}}}=1/(\sqrt{M}|\alpha |)$ is optimal, namely, the QCRB is calculated by Eq. (12). This bound is also called SQL. For comparison, we adopt the same measurement scheme with the MLE approach to evaluate the performance.

**Figure 2:**
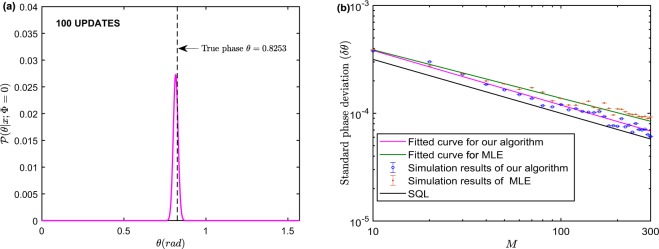
Monte Carlo simulation for coherent states with Algorithm 1 by setting the parameters *η*_*a*_ = *η*_*b*_ = 1, *λ* = 0, ${\mathscr{P}}(\theta )=2/\pi$ compared with HD by using MLE. (**a**) The posterior probability distribution of phase shift are sharp with the measurement times *M* = 100 when |*α*|^2^ = 100. (**b**) The standard phase deviation *δθ* varies with the number of measurement times *M* when |*α*|^2^ = 10^6^. Green and pink lines are fitted by the first typer of power function *y* = *ax*^*b*^. Each trial is simulated with 10^4^ times, and error bars are shown only if they are larger than the size of markers.

We first simulate Algorithm 1 with Monte Carlo method to evaluate the performance compared with the MLE approach. For simplicity, we set the transmissivity ${\eta }_{a}={\eta }_{b}=1,\lambda =0$ and prior distribution $\pi (\theta )=2/\pi$. When $|\alpha {|}^{2}=10$, the dynamic process of Algorithm 1 estimating the phase shift is also presented. The posterior probability is sharp while the measurement trials increase to 100 as Fig. 2(a) shows, which indicates that we accept the estimated phase with high confidence. When $|\alpha {|}^{2}={10}^{6}$, our algorithm and MLE can both approach the SQL well as the pink and green fitted line in Fig. 2(b) shows. These two lines are fitted by the first type power function $y=a{x}^{b}$ since it fits the expression of ${(\delta \theta )}_{{\rm{opt}}}$. It demonstrates that MLE and our algorithm can both saturate the SQL given a classical input. In particular, our algorithm still presents an advantage of efficiency in terms of the convergence rate given the same measurement trials. That is, our algorithm presents a faster convergence rate for a given precision. Consequently, we can conclude that by using MLE and our algorithm respectively, the precision saturates the SQL for the coherent state and moreover, our algorithm presents an advantage of efficiency in terms of convergence rate given the same measurement trials.

#### CSV state without feedback

In this part, we discuss the performance of Algorithm 1 under input of quantum CV states. It is believed that given one port be coherent state, the optimal state which can achieve the HL scaling for another port of MZI is squeezed vacuum state^24,44,45^. CSV state can be denoted as $|{\rm{CSV}}\rangle =|s\rangle \otimes |\alpha \rangle$, where $|s\rangle =S(r)|0\rangle$ is the squeezed vacuum state with $S(r)=\exp [r/2({\hat{a}}^{\dagger 2}-{\hat{a}}^{2})]$, and *r* is the squeeze parameter. *S*(*r*) can be represented by a linear transformation given by $S(r)={\rm{diag}}\{{e}^{r},{e}^{-r}\}$. The covariance matrix of squeeze vacuum state $V=S(r)S{(r)}^{T}={\rm{diag}}\{{e}^{2r},{e}^{-2r}\}$. Therefore, the first-order moment and covariance matrix of the input state are given by ${m}_{{\rm{in}}}^{{\rm{CSV}}}={\{|\alpha |,0,0,0\}}^{T},{{\rm{\Sigma }}}_{{\rm{in}}}^{{\rm{CSV}}}=\frac{1}{4}{\rm{diag}}\{1,1,{e}^{2r},{e}^{-2r}\}$. After CSV state propagating through the MZI and using HD for optimal phase quadrature $\hat{x}$ of the second output port, the mean and variance are given by (here we assume Φ = 0, *σ* = *∞*),

15 $${m}_{{\rm{o}}}^{{\rm{CSV}}}=\frac{1}{2}|\alpha |\,\sin \,\theta ,$$

16 $${({\sigma }_{{\rm{o}}}^{{\rm{CSV}}})}^{2}=\frac{1}{32}{e}^{-2r}(2{e}^{4r}{\sin }^{2}\theta -8{e}^{r}\,\sinh \,r\,\cos \,\theta +4{e}^{2r}+\,\cos (2\theta )+3).$$

It is readily to find that the minimum variance is taken at *θ* = 0 and equals to $\frac{1}{4}{e}^{-2r}$. The probability of obtaining quadrature $\hat{x}$ is given by,

17 $${{\mathscr{P}}}_{{\rm{C}}{\rm{S}}{\rm{V}}}(x|\theta ;{\rm{\Phi }})=\frac{1}{\sqrt{2\pi {({\sigma }_{{\rm{o}}}^{{\rm{C}}{\rm{S}}{\rm{V}}})}^{2}}}\exp (-\frac{{(x-{m}_{{\rm{o}}}^{{\rm{C}}{\rm{S}}{\rm{V}}})}^{2}}{2{({\sigma }_{{\rm{o}}}^{{\rm{C}}{\rm{S}}{\rm{V}}})}^{2}}).$$

By Eq. (9), we figure out the phase sensitivity determined by the phase difference for *M* number of repeated measurements given by,

18 $$\delta \theta =\sqrt{\frac{2{e}^{4r}{\sin }^{2}\theta -8{e}^{r}\,\sinh \,r\,\cos \,\theta +4{e}^{2r}+\,\cos \,2\theta +3}{8M|\alpha {|}^{2}{e}^{2r}{\cos }^{2}\theta }}.$$

The VTB is achieved at $\theta =k\pi ,k\in {\mathbb{Z}}$ (called sub-shot noise limit) for which ${(\delta \theta )}_{{\rm{opt}}}^{{\rm{CSV}}}={e}^{-r}/\sqrt{M}|\alpha |$. By Eq. (12), we calculate the QCRB ${(\delta \theta )}_{{\rm{Q}}}^{{\rm{CSV}}}={[M(|\alpha {|}^{2}{e}^{2r}+{\sinh }^{2}r)]}^{-1/2}$. When $|\alpha {|}^{2}\gg {\sinh }^{2}r$ (i.e. sin h^2^*r* can be neglected), the sub-shot noise limit is approximately equal to the HL. Given the total average photon number and if $|\alpha {|}^{2}\,\approxeq \,{\sinh }^{2}r$ (i.e., sin h^2^*r* cannot be neglected), the optimal phase sensitivity is achieved by parity or photon number detection^24,34^. In particular, photon number detection can avoid the dependence of phase estimation accuracy on the true phase value because it fully utilizes the statistical feature of each photon. However, these measurement methods for Heisenberg scaling are not implemented readily in practical quantum physical systems and more significantly, they are more sensitive to photon loss in lossy MZI^46,47^.

**Figure 3:**
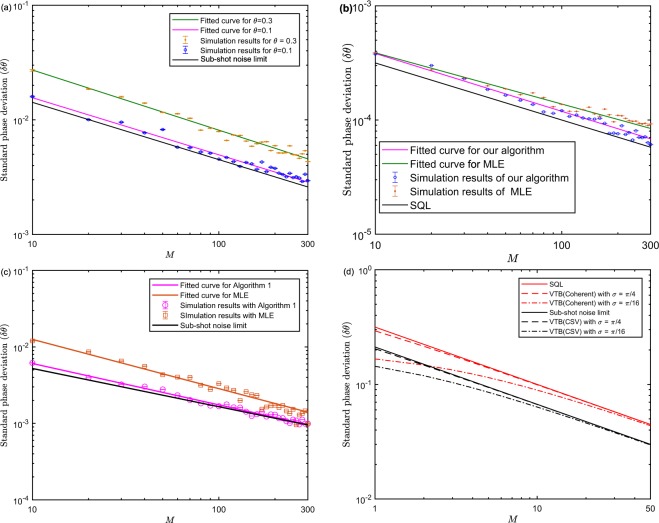
Simulation results for CSV probe states via Algorithm 1 with ${\eta }_{a}={\eta }_{b}=1,\lambda =0,{\mathscr{P}}(\theta )=2/\pi$. (**a**) The true phase shift sets to 0.1 and 0.3, respectively. The pink and green curves are fitted by the power function. The true phase shift sets to be optimal for HD with MLE (brown) and our algorithm (pink) for (**b**,**c**), and $|\alpha {|}^{2}=100,r=0.8$ for (**b**), $|\alpha {|}^{2}=100$, *r* = 1.8 for (**c**). The pink and green curves are fitted by the first type of power function *y* = *ax*^*b*^. Each trial is simulated with 10^4^ times and error bars are shown only if they are larger than the size of markers (**d**). The enhancement of VTB with respect to coherent (blue) and CSV (yellow) state compared with conventional bound.

Similarly, we still adopt the MLE as a comparison to illustrate the superiority of our algorithm. The standard phase deviation with CSV as input can be figured out for different number of measurements when $|\alpha {|}^{2}=100,r=0.8,1.8$ as Fig. 3(a–c) shows, in which all the lines are fitted by the first type of power function $y=a{x}^{b}$ for which is consistent with the form of ${(\delta \theta )}_{{\rm{opt}}}^{{\rm{CSV}}}$. Here, we set the parameters $\lambda =0,{\eta }_{a}={\eta }_{b}=1,{\mathscr{P}}(\theta )=2/\pi$.

In our physical model if we set *λ* = 0, HD without feedback control cannot eliminate the phase dependence of estimation on true phase shift, thus the phase sensitivity relies on the true phase shift in the whole interval of [0, *π*/2]. As Fig. 3(a) implies, when *θ* = 0.1, 0.3, respectively, the standard phase deviation *δθ* changes obviously and small phase shift, i.e. near the optimal phase, will have a better performance. Because if phase shifts severe, the measured fluctuation will be large, which will cause the effectiveness of squeeze operation to enhance precision being not achieved. In addition, we simulate our algorithm and HD with MLE, respectively as Fig. 3(b,c) shows. When $|\alpha {|}^{2}=100,r=0.8$ and the phase shift is set to be optimal, we find that HD with MLE cannot saturate sub-shot noise limit (near QCRB) in a few trials ($M < 300$). In particular, when *r* increases, we find that the gap between the sub-shot noise limit and the simulation results of HD with MLE becomes larger in the early stage of training. It indicates that MLE needs more samples to calculate the likelihood function and then estimate the optimal phase parameter. When the samples size is large, our algorithm will have nearly the same performance with MLE. Our algorithm saturates the sub-shot noise limit efficiently whatever *r* is larger or small. However, MLE shows a relatively slow convergence rate when *r* is small since small *r* indicates the larger fluctuations of output state. In conclusion, our algorithm shows a notable performance when the input is a quantum CV state. More specifically, our algorithm can saturate the sub-shot noise limit (when $|\alpha {|}^{2}\gg 1$, near QCRB or HL) efficiently compared with HD by using MLE when the input is CSV state in a situation of limited measurement trials.

#### Prior information analysis

In this part, we will compare the precision of existing uniform and different width of Gaussian prior distributions under a given phase shift for different probe states. In general, we assume a Gaussian prior of width *σ* > 0 centered at *θ* = *θ*_*p*_, that is,

19 $$\pi (\theta )=\frac{1}{\sqrt{2\pi {\sigma }^{2}}}\exp \{-\frac{{(\theta -{\theta }_{p})}^{2}}{2{\sigma }^{2}}\},$$

with mean value *θ*_*p*_ and variance *σ*^2^. The Fisher information of *π*(*θ*) can be calculated by Eq. (9) for which ${\mathcal I} [\pi (\theta )]={\sigma }^{-2}$. Thus, the VTB for coherent input states with *M* repeated HDs as Fig. 1 depicted is given by,

20 $${{\mathscr{V}}}_{c}^{2}[{\rm{\Theta }}({\mathscr{X}});\pi ]\ge \frac{1}{M|\alpha {|}^{2}+{\sigma }^{-2}}.$$

Note that the width of Gaussian prior *σ* needs to be restricted within an interval since a large width (variance) will cause the MSE rule not working well for phase estimation^40^. Eq. (20) demonstrates that if we have much information on the phase shift before measurements, i.e., the width of $\sigma \ll 1$, the precision will be highly improved. Similarly, if the CSV states are the input, the VTB with *M* repeated HDs is given by,

21 $${{\mathscr{V}}}_{s}^{2}[{\rm{\Theta }}({\mathscr{X}});\pi ]\ge \frac{1}{M|\alpha {|}^{2}{e}^{2r}+{\sigma }^{-2}}.$$

We plot the VTB for *M* repeated measurements with respect to three different Gaussian width $\sigma =\infty ,\pi /4,\pi /16$ when $|\alpha {|}^{2}=10$ for coherent state and $|\alpha {|}^{2}=10,r=0.8$ for CSV state as Fig. 3(d) shows. Theoretically, the VTB is superior to the CRB with *a prior* information *σ* according to Eqs (20–21). As expected, the improvement will gradually vanish when measurement times *M* increases since *σ*^−2^ can be neglected compared with the Fisher information of the posterior distribution. However, when *M* ≤ 10, the enhancement of *δθ* is obvious with a small Gaussian width. Therefore, the VTB can adapt to the situation where the measurements are extremely limited and the prior information is provided.

**Figure 4:**
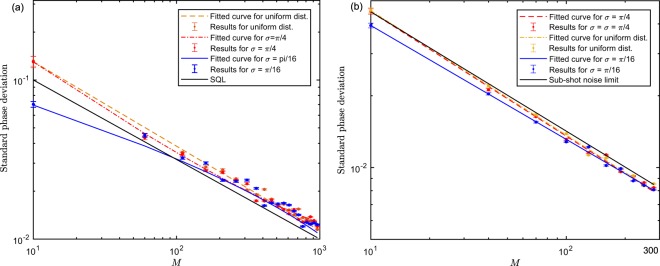
Phase estimation with different Gaussian prior width *σ* via Algorithm 1 for $\eta =1,\lambda =0$, as a function of measurement trials *M*. The fitted curves adopt the second type of power function *y* = *ax*^*b*^ + *c* as the fitting function since the prior information *σ* is an invariant with *M* as Eq. (20,21) indicated. Monte Carlo simulation parameters of $|\alpha {|}^{2}=10$, $\theta =0.8253$, *M* = 1000 set for left panel of the graph, and $|\alpha {|}^{2}=10,r=0.8,\theta =0.2352,M=300$ for right panel of the graph.

Without loss of generality, we will set a relatively small phase shift to analyze the impact of prior knowledge on the ultimate estimated precision. For simplicity, we will not consider the case of phase ambiguity, thus the feedback control will not be adopted i.e., *λ* = 0. If the probe states are CSV states, we know that the effect of phase shift on precision has been eliminated via feedback control. In Fig. 4(a,b), the fitted lines are fitted by the second type of power function *y* = *ax*^*b*^ + *c* on account of the invariant impact of prior information playing the role of constant value *c* when *σ* = *π*/4, *π*/16. It is found that the second type of power function has a better fitting fidelity in the simulation since the goodness of fit coefficient *R*^2^ = 0.9923 is larger than the situation where the first type of power function is employed whose *R*^2^ = 0.9732 when the input state is the coherent state. Similarly, when the input state is CSV state, the goodness of fit coefficient *R*^2^ = 0.9923 of the second type of power function is larger than the first type whose *R*^2^ = 0.9852 as expected. The difference is not such significant since the prior information *σ* reduces smaller quantum fluctuations compared to the input being the coherent state. We find that the Gaussian prior knowledge can certainly enhance the precision for coherent and CSV state with small measurement trials (*M* < 100), but its impact vanishes when trial becomes large because our algorithm uses the history measurement information to update the prior distribution, which also validates the above theoretical analysis. In particular, the improvement when *σ* = *π*/4 is not greater than *σ* = *π*/16. Therefore, smaller width will have a better performance improvement on precision. The precision of phase estimation can be reached more efficiently when given the prior information.

#### Feedback strategy and photon loss analysis

In general, if the phase shift is limited to the interval [0, *π*/2], our algorithm with *λ* = 0 performs well both for coherent and CSV state as described above. However, in order to eliminate phase ambiguity in [0, 2*π*] which exists in many CV quantum phase estimation problems^48^, we present an explicit feedback strategy which can extract information^49^ about phase shift from each measurement. For *k*th measurement, we define the information feedback function explicitly for ${\rm{\Gamma }}({{\mathscr{X}}}_{k})$ which is given by,

22 $${\theta }_{\ast }^{(k)}={{\rm{\Phi }}}^{(k)}-\arcsin (\frac{2{x}_{k}}{|\alpha |})\zeta (\frac{|\alpha |}{2}-|{x}_{k}|)-\frac{\pi }{2}{\rm{s}}{\rm{g}}{\rm{n}}({x}_{k})\zeta (|{x}_{k}|-\frac{|\alpha |}{2}),$$

where $\zeta (z)=1,z\ge 0$, $\zeta (z)=0,z < 0$, and sgn(·) is the sign function. Eq. (22) denotes that an effective phase feedback occurs when the measured quadrature phase is less than the half of squared average photon number, otherwise executing *π*/2 rotation in an opposite direction. It is observed that large quantities of HD outcomes *x*_*k*_ satisfy the condition of *x*_*k*_ > |*α*|/2, and the second case for which *x*_*k*_ > |*α*|/2 is not relevant for highly displaced coherent input states, where such events become very rare. If we set the threshold value of |*α*|/2 even larger, then more samples will be collected to calculate the feedback phase information. This may speed-up the convergence of the feed-back phase to the true phase but is at the cost of reducing the robustness especially in the final stage. The corespondent probability distribution of *x*_*k*_ > |*α*|/2 for coherent and CSV state is given by,

23 $${{\mathscr{P}}}_{c}({\theta }_{\ast }^{(k)})=\frac{1}{2}{\rm{erfc}}[\frac{|\alpha |}{\sqrt{2}}(1\pm \,\sin ({\varepsilon }^{(k)}))],$$

24 $${{\mathscr{P}}}_{s}({\theta }_{\ast }^{(k)})=\frac{1}{2}{\rm{erfc}}[\frac{\sqrt{({\sinh }^{2}r+|\alpha {|}^{2})}}{{\sigma }_{{\rm{o}}}^{{\rm{CSV}}}({\varepsilon }^{(k)})}(1\pm \,\sin ({\varepsilon }^{(k)}))],$$

where ${\rm{erfc}}(x)=\frac{2}{\sqrt{\pi }}{\int }_{x}^{\infty }\exp (\,-{t}^{2}){\rm{d}}t$ denotes the complementary error probability function. Eq. (23) denotes the probability that we cannot extract effective phase information. Here, we provide a heuristic way of trying to extract true phase information among the measured samples. This strategy is a tracking process such that the feedback phase will be updated towards to the true phase shift when one obtains the measurement results. Thus, the true phase shift *θ* is theoretically broadened to the whole interval of [0, 2*π*] since the feedback phase can always be adjusted to track the unknown phase shift.

**Figure 5:**
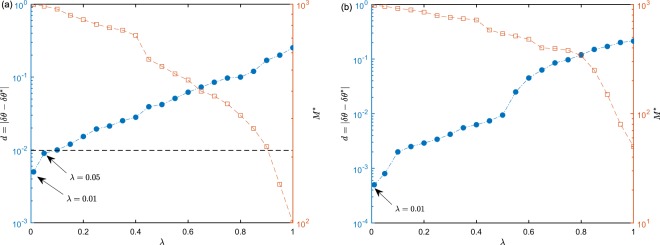
Optimization of the learning rate *λ*. (**a**) For the coherent state, the left blue axis is plotted by executing Algorithm 1 when given $|\alpha {|}^{2}=10,M={10}^{3}$. The right yellow axis is plotted with $|\alpha {|}^{2}=10$. (**b**) For CSV state, the left blue axis is plotted with $|\alpha {|}^{2}=10,r=0.8,M={10}^{3}$. The right yellow axis is plotted with $|\alpha {|}^{2}=10,r=0.8$.

In addition, the learning rate *λ* in our algorithm as a hyperparameter needs to be optimized otherwise will cause the posterior distribution not converged or converged to a wrong position. Intuitively, $\lambda \to 0$ is equivalent to the case of no feedback control, which thus will render the posterior converge to an ideal position by using a large *M*. However, the dynamical range of phase estimation cannot be broadened under this parameter setting. When *λ* → 1, the posterior distribution will converge fast to a wrong position only by using fewer trials *M*. Therefore, we need a trade-off between the convergent trials M* and error precision $d=|\delta \theta -\delta {\theta }^{\ast }|$, where δθ* is the ideal precision calculated by VTB. A reasonable rule for the acceptance is that *d* < δθ*. In order to optimize *λ*, we test the error precision *d* and M* under a set of different value of *λ* for the coherent and CSV state, respectively. For the coherent state, we set $|\alpha {|}^{2}=10,{\eta }_{a}={\eta }_{b}=1,M={10}^{3}$ in Algorithm 1 to test the error precision *d* with different learning rate *λ*. As Fig. 5(a) shows, the error precision preserves a small and an acceptable value when $\lambda =0.01,0.05$ and M^*^ approximately equal to *M*, which indicates that the posterior distribution converges to a correct position. For larger *λ*, *d* is unacceptable although the convergent trials M^*^ becomes smaller. Similar for CSV state, we set $|\alpha {|}^{2}=10,r=0.8,{\eta }_{a}={\eta }_{b}=1,M={10}^{3}$ to test the error precision under different value of *λ*. In Fig. 5(b), the error precision maintains a similar feature with the coherent state and are acceptable when $\lambda =0.01,{M}^{\ast }\approx {10}^{3}$, which demonstrates that the phase precision is still approximately optimal and not reduced by the feedback learning. Moreover, it is found that *M* = 10^3^ is matched with the learning rate *λ* = 0.01 both for the coherent and CSV state. Larger *M* will also be appropriate for *λ* = 0.01 for which can render posterior distribution converged correctly, i.e., M* = 10^3^ is the minimum measurement trials for *λ* = 0.01.

**Figure 6:**
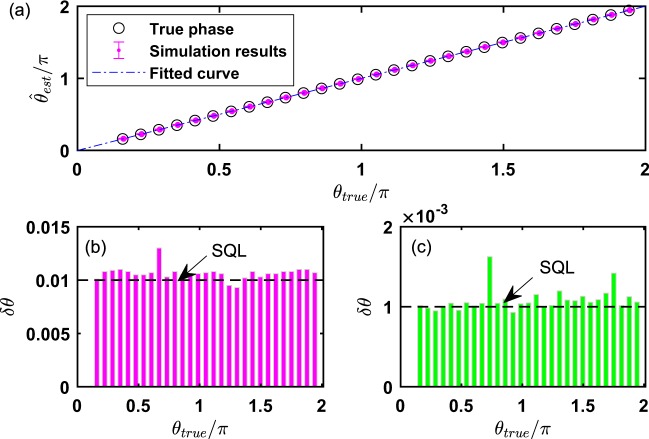
The phase ambiguity of coherent state is eliminated via Algorithm 1 with feedback strategy ${\rm{\Gamma }}({\mathscr{X}})$ by setting ${\mathscr{P}}(\theta )=1/2\pi ,{\eta }_{a}={\eta }_{b}=1,M={10}^{3},\lambda =0.01$. (**a**) The true phase and estimated phase are plotted with $|\alpha {|}^{2}=10$ and error bars are shown only if they are larger than the size of markers. The bars of (**b**,**c**) are simulated with $|\alpha {|}^{2}=10,{10}^{3}$, respectively.

**Figure 7:**
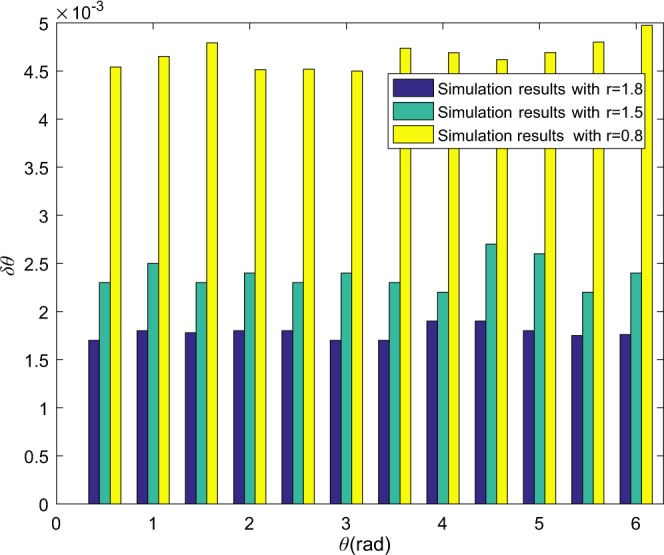
The phase ambiguity is eliminated via Algorithm 1 with ${\rm{\Gamma }}({\mathscr{X}})$. The three kinds of bars denote the standard phase deviation *δθ* with respect to $r=1.8,1.5,0.8$ for true phase shift in [0, 2*π*]. The simulation parameters are set to be $|\alpha {|}^{2}=10$, $M={10}^{3}$, $\lambda =0.01,{\eta }_{a}={\eta }_{b}=1$.

We analyze two cases for classical input where $|\alpha {|}^{2}=10,{10}^{3}$ respectively and the phase shift *θ* ∈ [0, 2*π*] via Algorithm 1 with feedback function ${\rm{\Gamma }}({\mathscr{X}})$ as Fig. 6 shows. The optimal choice of *λ*, M* can be appropriate for the case of large photon numbers since *d* is not directly steered by *α*. Therefore, we set the parameters ${\eta }_{a}={\eta }_{b}=1,{\mathscr{P}}(\theta )=1/2\pi ,\lambda =0.01$ to analyze the performance with the feedback strategy. The results show that for arbitrary phase shift, the precision saturates the SQL as Fig. 6(a–c) shows regardless of small or large $|\alpha {|}^{2}$. Thus, it demonstrates that Algorithm 1 with feedback function ${\rm{\Gamma }}({\mathscr{X}})$ can eliminate the phase ambiguity of CV phase estimation in [0, 2*π*]. In order to eliminate the impact of true phase shift on the precision of parameter and phase ambiguity for CSV state, we adopt the same feedback strategy as the coherent state does. The precision performance will not be steered by the true phase shift via Algorithm 1 with the proposed feedback strategy ${\rm{\Gamma }}({\mathscr{X}})$ if we can eliminate the phase ambiguity since the feedback phase can always be adjusted towards the true phase. We simulate Algorithm 1 with the phase shifts sampled from the interval of [0, 2*π*] with $|\alpha {|}^{2}=10,r=0.8,1.5,1.8$ and measurement trials $M={10}^{3}$ for $\lambda =0.01$, ${\eta }_{a}={\eta }_{b}=1$ as Fig. 7 shows. The three bars with respect to different squeeze parameter basically reach their sub-shot noise limit ${\delta }_{{\rm{Sub}}-{\rm{shotnoise}}}=0.0045,0.0022,0.0017$, which demonstrates that the phase ambiguity is also eliminated for CSV state. Our feedback strategy is an explicit function and need not complex optimization process to obtain the feedback information compared with ref.^26^. Thus, it can be extended to many real-time phase estimation scenarios.

In our algorithm, we mainly consider the linear photon loss caused by photon loss inside the interferometer and photon loss due to the inefficient detector. Specifically, assuming the losses are existed in two arms and inserted between the first BS and phase shifter^47^. Here, the photon losses are symmetric in two arms, i.e., $\eta ={\eta }_{a}={\eta }_{b}$, and we aim to validate whether our algorithm is robust against linear photon losses. The mean and variance value of the coherent state as input in terms of symmetric lossy MZI are calculated with detection of quadrature $\hat{x}$ of the second port for which ${m}_{\eta }^{{\rm{o}}}=1/2\sqrt{\eta }|\alpha |\,\sin \,\varepsilon ,{({\sigma }_{\eta }^{{\rm{o}}})}^{2}=1/4.$ Thus, we can calculate the VTB with *a prior* information *σ* under symmetric linear photon loss for *M* repeated measurements given by,

25 $${(\delta \varepsilon )}_{C}^{{\rm{loss}}}=\sqrt{\frac{1}{M\eta |\alpha {|}^{2}+{\sigma }^{-2}}}.$$

The optimal phase difference is $${\varepsilon }_{{\rm{opt}}}^{C}=k\pi ,k\in {\mathbb{Z}}$$. Eq. (25) can also be calculated by Eq. (12), implying HD is optimal under linear photon loss. Similarly, for CSV state, we have $${m}_{\eta }^{{\rm{oCSV}}}=\frac{1}{2}\sqrt{\eta }|\alpha |\,\sin \,\varepsilon ,{({\sigma }_{\eta }^{{\rm{oCSV}}})}^{2}$$$$=\frac{1}{16}(-3\eta +\eta {e}^{2r}{\sin }^{2}\varepsilon +4\eta {e}^{-2r}{\cos }^{4}(\frac{\varepsilon }{2})-\eta \,\cos \,\varepsilon +4).$$ The VTB of CSV state as input with *a prior* information *σ* under symmetric linear photon loss for *M* repeated measurements is given by,

26 $${(\delta \varepsilon )}_{{\rm{CSV}}}^{{\rm{loss}}}=\sqrt{\frac{\eta {e}^{-2r}+1-\eta }{M\eta |\alpha {|}^{2}+(\eta {e}^{-2r}+1-\eta ){\sigma }^{-2}}}.$$

The optimal phase difference $\varepsilon =k\pi ,k\in {\mathbb{Z}}$. Note that ${(\delta \varepsilon )}_{{\rm{CSV}}}^{{\rm{loss}}}=\sqrt{1/M|\alpha {|}^{2}{e}^{2r}+(1-\eta )/M\eta |\alpha {|}^{2}}$ when the prior distribution is uniform, which implies that if we increase |*α*|^2^ (coherent power), the effects of photon loss will be suppressed dramatically and ultimately the phase precision approaches to the sub-shot noise limit. The QCRB of CSV state as input in a lossy MZI without prior information for *M* repeated measurements can be calculated by Eq. (12) given by,

$${(\delta \varepsilon )}_{Q}^{{\rm{loss}}}=\sqrt{\frac{(1-\eta )({e}^{2r}-1)+1}{M\eta \{|\alpha {|}^{2}{e}^{2r}+{\sinh }^{2}r[(1-\eta )({e}^{2r}-1)+1]\}}},$$

an equation that can also be found in the literature^50^. We find that when $|\alpha {|}^{2}\gg 1$ and $\sigma =\infty ,$ ${(\delta \varepsilon )}_{{\rm{CSV}}}^{{\rm{loss}}}\,\approxeq \,{(\delta \varepsilon )}_{Q}^{{\rm{loss}}}$.

**Figure 8:**
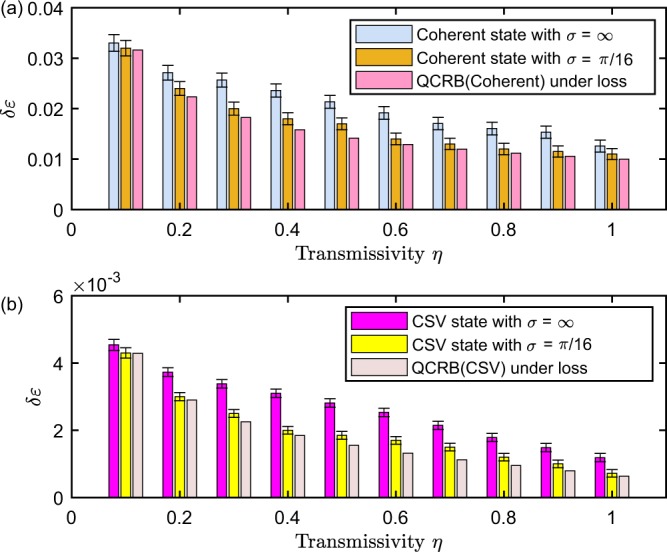
Influence of different transmissivity $\eta$ on parameter estimation precision. The input parameters $\lambda =0.01$, $M={10}^{3}$, ${\eta }_{a}={\eta }_{b}$, $\sigma =\infty ,\pi /16$. (**a**) The blue bars denote the simulation results for the coherent state with $|\alpha {|}^{2}=10,\sigma =\infty$ via Algorithm 1 with ${\rm{\Gamma }}({\mathscr{X}})$, and the orange bars are the simulation results with $|\alpha {|}^{2}=10,\sigma =\pi /16$ via Algorithm 1 with ${\rm{\Gamma }}({\mathscr{X}})$ and the pink bars display the QCRB under the condition of linear photon loss with the flat prior distribution calculated by Eq. (25). (**b**) The red bars are the simulation results for CSV state with $r=0.8,|\alpha {|}^{2}=10,\sigma =\infty$, and the yellow bars denote the simulation results for CSV state with $r=0.8,|\alpha {|}^{2}=10,\sigma =\pi /16$, and the pink bars are the QCRB in linear lossy MZI with the flat prior distribution, respectively.

We discuss the impact of transmissivity *η* of BS on phase estimation by Algorithm 1 with ${\rm{\Gamma }}({\mathscr{X}})$ when given different prior information with respect to the coherent and CSV state. As Fig. 8(a) demonstrates, compared to the case of no prior information assisted, the phase deviation *δε* nearly saturates the VTB (QCRB) in lossy MZI when given the prior information *σ* = *π*/16, which indicates that *a prior* integrated algorithm can enhance the robustness of the phase estimation. From Fig. 8(b), compared to the case of *σ* = *∞*, the standard deviation *δε* of the simulation results basically saturates the QCRB when given the prior information *σ* = *π*/16. In particular, for small *η*, the performance is significantly superior to the situation where no prior information is employed. When *η* becomes large, the enhancement are not such significant, which conversely implies that *a prior* information can enhance the robustness of the algorithm. As a result, it is implied that the standard phase deviation can saturate the lossy QCRB for the coherent and CSV state by executing Algorithm 1with feedback strategy when given the prior information, which also demonstrates that the effect of linear photon loss on the phase estimation precision can be suppressed dramatically.

## Conclusions

We investigate a quantum phase estimation framework for Gaussian states based on a machine learning approach. We incorporate the recursive Bayesian estimation method and an explicit feedback strategy to the algorithm organically, which renders it more flexible in phase estimation tasks. We have studied two probe practical Gaussian resources that are available in the laboratory and easily produced, for optical quantum metrology. Based on Algorithm 1, compared with the conventional scheme, it is efficient to approach the theoretical bound with feasible CV states. Moreover, by assuming a Gaussian prior being employed, we find that the precision enhancement corresponds to the different width *σ*, i.e. prior information assists the phase estimation being more efficient. In particular, the phase ambiguity of coherent and CSV state can be eliminated via Algorithm 1 with the proposed feedback strategy such as to achieve the physical limits with arbitrary phase shift in [0, 2*π*]. To validate the robustness of Algorithm 1, we analyze the lossy QCRB in MZI with symmetric linear photon loss for the coherent and CSV state, and the simulation results present negligible precision reduction compared with theoretical lossy QCRB especially for the case of *a prior* information integrated, which implies that the photon losses are suppressed dramatically by Algorithm 1 with our feedback strategy and prior information. Our CV quantum phase estimation framework highlights the machine learning method, studies the CV phase estimation and can be extended to the time-variable or multi-parameter estimation framework.

## Supplementary information

Continuous-variable Quantum Phase Estimation based on Machine Learning
