# Supplementary material for: Continuous-variable Quantum Phase Estimation based on Machine Learning
Source: Sci Rep. 2019 Aug 27;9:12410. doi: 10.1038/s41598-019-48551-0 (PMC6711976; doi:10.1038/s41598-019-48551-0)
Supplement: Supplementary file 1 — Continuous-variable Quantum Phase Estimation based on Machine Learning [file 41598_2019_48551_MOESM1_ESM.pdf]

Continuous-variable Quantum Phase Estimation based on Machine Learning

Tailong Xiao [1], Jingzheng Huang [1], Jianping Fan [2] and Guihua Zeng*[1]

[1]State Key Laboratory of Advanced Optical Communication Systems and Networks, and Center of Quantum Information Sensing and Processing, Shanghai Jiao Tong University, Shanghai 200240, China

[2]Department of Computer Science, University of North Carolina-Charlotte, Charlotte, North Carolina 28223, USA

July 11, 2019

Appendix

Calculation of first order and second order moment

Since the Hamiltonian of MZI should be Hermitian matrix, the evolution operators can be denoted by unitary matrices. After the input states propagate through the MZI, the covariance matrix Σ_{out} and first-order moment d_{out} of output state can be operated by $\Sigma_{\text{out}} = \Psi_{\text{MZI}}\Sigma_{\text{in}}\Psi_{\text{MZI}}^T, d_{\text{out}} = \Psi_{\text{MZI}}d_{\text{in}}$, where Σ_{in} and d_{in} are the covariance matrix and first-order moment of the input state. Ψ_{MZI} denotes the unitary operation of MZI. More generally, a lossy MZI can be described as $\Psi_{\text{MZI}} = \Psi_2\Psi_{\theta,\Phi}(\gamma \circ \kappa)\Psi_1$, where $\gamma \circ \kappa$ is the composition transformation with respect to covariance matrix and first-order moment in the photon loss channel. Thus, the covariance matrix and first-order moment of the output state are transformed as the following rule,

$$\Sigma_\eta^{\text{out}} = \Psi_2\Psi_{\theta,\Phi}\gamma(\Psi_1\Sigma\Psi_1^T)\Psi_{\theta,\Phi}^T\Psi_2^T, \tag{1}$$

$$d_\eta^{\text{out}} = \Psi_2\Psi_{\theta,\Phi}\kappa(\Psi_1 d), \tag{2}$$

where $\Sigma_\eta^{\text{out}}, d_\eta^{\text{out}}$ are the output covariance matrix and first-order moment for lossy MZI. $\Psi_{\theta,\Phi}$ represents the operation of phase shifter for two arms (modes).

*ghzeng@sjtu.edu.cn

Ψ_1 and Ψ_2 denotes the operation of first BS and second BS, respectively. The most general symplectic matrix with respect to BS is $\Psi(\phi)$ given by,

$$\Psi(\phi) = \begin{pmatrix} \cos\phi e^{i\beta_0} & 0 & -\sin\phi e^{-i\beta_1} & 0 \\ 0 & \cos\phi e^{-i\beta_0} & 0 & -\sin\phi e^{-i\beta_1} \\ \sin\phi e^{i\beta_1} & 0 & \cos\phi e^{-i\beta_0} & 0 \\ 0 & \sin\phi e^{i\beta_1} & 0 & \cos\phi e^{i\beta_0} \end{pmatrix}. \tag{3}$$

In special case of the first BS, $\phi = \pi/4$ and $\beta_0 = \beta_1 = 0$, the matrix is $\Psi(\pi/4)$. The second BS can be described with matrix $\Psi_2 = \Psi(-\pi/4), \beta_0 = \beta_1 = 0$. The symmetric phase shifter operation can be denoted with matrix $\Psi_{\theta,\Phi}$ given by,

$$\Psi_{\theta,\Phi} = \begin{pmatrix} \cos\theta & \sin\theta & 0 & 0 \\ -\sin\theta & \cos\theta & 0 & 0 \\ 0 & 0 & \cos\Phi & -\sin\Phi \\ 0 & 0 & \sin\Phi & \cos\Phi \end{pmatrix}, \tag{4}$$

where θ is the unknown phase shift, and Φ is the feedback control phase. Generally, fictitious BS which demonstrates linear photon loss can also be described with the symmetric matrix with $\phi = \arccos\sqrt{\eta}$, where η is the transmissivity of BS. Consequently, the covariance matrix and the first-order moment of two-mode Gaussian states can be transformed with transmissivity η_a, η_b for modes a and b in photon loss channel as [1]

$$\gamma(\Sigma) = D_1 \Sigma D_1 + D_2/2, \tag{5}$$

$$\kappa(d) = D_1 d, \tag{6}$$

where D_1 and D_2 are diagonal matrices denoted with $D_1 = \mathrm{diag}(\sqrt{\eta_a}, \sqrt{\eta_a}, \sqrt{\eta_b}, \sqrt{\eta_b}), D_2 = \mathrm{diag}(1-\eta_a, 1-\eta_a, 1-\eta_b, 1-\eta_b)$, respectively. Note that the output state is still Gaussian state after the transformation of the MZI.

References

[1] Oh, C., Lee, S.-Y., Nha, H. & Jeong, H. Practical resources and measurements for lossy optical quantum metrology. *Phys. Rev. A* **96**, 062304 (2017).
